# Secondary Outcomes of a Front-of-Pack-Labelling Randomised Controlled Experiment in a Representative British Sample: Understanding, Ranking Speed and Perceptions

**DOI:** 10.3390/nu14112188

**Published:** 2022-05-24

**Authors:** Jessica Packer, Simon J. Russell, Deborah Ridout, Anne Conolly, Curtis Jessop, Russell M. Viner, Helen Croker

**Affiliations:** 1 Population, Policy and Practice Research and Teaching Department, UCL Great Ormond Street Institute of Child Health, University College London, London WC1N 1EH, UK; s.russell@ucl.ac.uk (S.J.R.); d.ridout@ucl.ac.uk (D.R.); r.viner@ucl.ac.uk (R.M.V.); h.croker@ucl.ac.uk (H.C.); 2 National Center for Social Research, London EC1V 0AX, UK; anne.conolly@natcen.ac.uk (A.C.); curtis.jessop@natcen.ac.uk (C.J.)

**Keywords:** nutritional labelling, front-of-pack label, comprehension, randomised controlled experiment, nutrition policy

## Abstract

Front-of-pack labels (FOPLs) provide simplified nutritional information that aims to inform consumer choice and encourage reformulation. We conducted an online randomised controlled experiment on a representative British sample to test the effectiveness of FOPLs across a range of outcomes. The primary outcomes have been published; here, we present the secondary outcomes: the ability to rank the healthiest product and the time to complete the rankings by comparing the FOPL groups and a no-label control, as well as a descriptive analysis of the perceptions. Participants from the NatCen panel were randomised to one of five experimental groups (Multiple Traffic Lights; Nutri-Score; Warning Label; Positive Choice tick; no-label control). Six food/drink categories were selected (pizza, drinks, cakes, crisps, yoghurts, breakfast cereals), and three products were created with varying healthiness. The participants (analytic sample = 4530) were asked to rank the products in order of healthiness twice (baseline: no label; follow-up: experimental group label). Compared to the control, the probability of correctly ranking the healthiest product at follow-up was significantly greater for the N-S, MTL and WL across all products. The time to correctly complete the ranking was fastest for the N-S, PC and no-label control. The descriptive analysis showed that the FOPLs were perceived favourably, and especially N-S and MTL. The findings were supportive of the primary analyses, with those results suggesting that N-S performed the best, and then MTL.

## 1. Introduction

Front-of-pack labels (FOPLs) are simplified nutritional labels that are displayed on the front of food- and drink-product packaging. The aim of FOPLs is to provide simple and easy-to-understand information to consumers to aid healthier choices and to drive reformulation [1,2]. Consumer-friendly FOPLs that are easy to understand or interpretative have been recommended for use by the World Health Organisation (WHO) since 2014 [3]. There are many different FOPLs that are used globally, which vary by the level and type of information provided, the colour and the shape. Prominent labels include:

- Multiple Traffic Lights (MTL): This label provides nutrient-specific information and an interpretation of those amounts in colour (red = high, etc.);
- Nutri-Score (N-S): This label provides a summary and interpretation of the overall nutrition (A-E) with corresponding colours (A/green = healthier, etc.);
- Warning Label (WL): This label provides nutrient-specific binary warnings;
- Positive-choice-style labels (PC): This is an overall binary label (healthy or not healthy).

Evidence suggests that summary labels are easier for consumers to understand and interpret compared to nutrient-specific labels, and especially for at-risk populations [4,5].

The effectiveness of FOPLs at equipping consumers with information to guide healthier choices relies on the ability of consumers to accurately understand the information, and then to use that information when making purchase and consumption decisions [6]. Grunert and Wills present a theoretical framework for how FOPLs impact consumer responses, and they outline that consumers need to see, understand and like the labels. They also specify modifying factors for label effectiveness, including demographic characteristics, the label format and interest or knowledge in nutrition [7]. For labels to be seen, which is the first step in the framework, FOPLs need to be shown on all products (e.g., mandatory implementation). Other factors that have been shown to impact actual use include familiarity, credibility and trust in the accuracy of the label; the context (including time pressures, price, marketing, product claims); product preferences; and previous purchases [8,9]. 

Experimental studies show that FOPLs are effective at improving the ability of participants to identify and rank products according to healthiness [10,11,12]. Evidence suggests that interpretive labels (i.e., MTL and N-S) are most effective for impacting consumer behaviours, and specifically labels that provide a simple evaluation of a product or that use colour for interpretation [2,13,14,15,16,17]. We have previously shown, in an online experimental study with a representative British sample, that all FOPLs were effective at improving the participants’ ability to correctly rank products according to healthiness [18]. N-S appeared to perform the best at improving knowledge, followed by MTL, but we still do not know which labels consumers could use correctly the quickest, the perceptions of the FOPLs or whether the finding for ranking just the healthiest product differed compared to ranking all three products. The correct ranking of the healthiest product is especially relevant for PC, as it is a binary label and, therefore, it is not possible to correctly rank three products (the primary outcome for the study). 

An analysis of FOPL policy across the world identified that 32 governments endorse FOPLs, of which 10 have made them mandatory [19]. Currently, FOPLs are not mandatory in the United Kingdom, which is due to its previous European Union membership (which does not permit/constrain mandatory labels) [20]. The UK government is committed to updating the FOPL policy on the basis of evidence so that it is the “best for Britain” [21,22]. The ability to correctly understand the comparative healthiness of products is important, and it was the primary outcome of our previously published [18] experiment. However, other factors are also important, and especially those that impact on actual use and that affect the likely effects of the FOPL policy. 

In this paper, we present the secondary analyses from our previously published experimental study. We examine whether FOPLs were effective at improving the participant understanding of the healthiest product; whether the time taken to complete the ranking tasks differed between FOPLs; and the participants’ perceptions of the FOPLs. Our main objectives were: (1) to identify if FOPLs (MTL, N-S, WL, PC) improved the participants’ ability to rank the healthiest product to a greater extent than the no-label control; (2) to identify if FOPLs differ in the time taken for participants to complete the ranking tasks; and (3) to descriptively examine the participant perceptions of the FOPLs. Consistent with previous analyses, we directly compared N-S and MTL, since evidence suggests that these are the best-performing labels.

## 2. Materials and Methods

The materials and methodology of this experiment have been detailed elsewhere [18]. Below, we provide a brief summary of the design.

### 2.1. Study design and Participants

This was an online randomised experimental study that tested five experimental conditions (MTL, N-S, WL, PC, no-label control). Participants from the NatCen panel were recruited to take part in an online experiment. The NatCen panel is nationally representative of Great Britain (GB) and is a probability-based sample. To take part in the experiment, the participants were required to be adults (aged 18 years or older), to be able to read and write in English and to complete the survey online. All active panel members were invited by email, post or SMS to participate, and they were offered a small financial incentive for completing the experiment. Data collection occurred between 28 October and 15 November 2020. Ethical approval was granted by NatCen’s Research Ethics Committee (application reference: P15640).

### 2.2. Materials

Six food and drink categories were selected (pizza, instant hot chocolate, cake, crisps, yoghurt, breakfast cereal). Three mock products were created with varying nutritional compositions for each category: a “most healthy”, an “in between” and a “least healthy”. Mock images of the products were created by a graphic designer, and no nutrition or quality claims were included (gluten free, high in protein, etc.). Full details of the study protocol are available on Open Science Framework (https://osf.io/k9v2p/, accessed 1 January 2022). 

### 2.3. Randomisation

Randomisation was stratified on the basis of key variables previously collected by NatCen: the year of recruitment to the panel; sex; age; government-office region; and household income. SPSS (Version 26.0, IBM Corp, Armonk, NY, USA) was used by NatCen to randomly allocate equal numbers of participants to each of the five experimental groups (MTL, N-S, WL, PC or control). The allocation was concealed from participants and researchers. 

### 2.4. Procedure

#### 2.4.1. Ranking Task

Participants viewed images of three products and were asked to rank them in order of healthiness, from most to least healthy. Participants completed the ranking tasks once with no FOPLs (baseline ranking tasks), and then again with their assigned FOPL shown on the products (follow-up ranking tasks). A total of 12 ranking tasks were completed by each participant (six product categories by two ranking conditions: baseline and follow-up). Participants were asked if they had enough information to rank the products after each task at both baseline and follow-up (12 occasions), and how confident they were after each set (baseline and follow-up, two occasions). Participants were able to zoom in on the image, but no other information was presented (including no back-of-pack information). To minimize missing data, participants could only select “don’t know” after attempting to move to the next page without completing the ranking task, and software prevented ranking two products the same. The presentation of categories and products within categories was randomised. The time taken to complete rankings was taken from the survey software, which recorded the length of time (seconds) spent on each web page (each ranking task was on a separate page). Participants were advised to click next as soon as they had completed each task (see Supplementary Table S1 for full questionnaire). The healthiest-product outcomes and the speed-of-ranking outcomes were based on the ranking tasks. None of the FOPLs were introduced or explained to participants.

#### 2.4.2. Label Perceptions

After the follow-up ranking tasks, to check that labels had been seen, participants were shown an image of their label condition and were asked if they had seen the FOPL. They were then asked if they had used the FOPL to complete the rankings, how easy/difficult they found the labels to understand, if they wanted the labels on packaging in the United Kingdom, how helpful in choosing what to buy they would be and views on how long it takes to use the labels. These questions were not applicable to the control group, as they did not see any FOPLs.

### 2.5. Measures

The primary outcome of the study was the ability to correctly rank products according to healthiness, the results of which have been published [18]. The secondary outcomes of the experiment presented in this paper are detailed below.

#### 2.5.1. Secondary Outcomes

Healthiest-product ranking outcomes were examined in three ways:

1. Ranking of the healthiest product (correct = 1, incorrect = 0) at baseline and follow-up;
2. Change in ranking from baseline to follow-up (improved = +1, no change = 0, worsened = −1);
3. Change in healthiest-product global food score, aggregated change in rankings for the five food products (score range was from –5 to +5).

Speed of ranking:

4. Time to complete each ranking (seconds) at baseline and follow-up.

#### 2.5.2. Descriptive Outcomes

Label perceptions, specific to the participants’ label conditions (control group was excluded):

5. Saw the label in the ranking tasks—dichotomised as yes vs no/not sure;
6. Used the label in the ranking tasks—all/some/did not use;
7. Ease of label understanding—dichotomised as “easy” (very easy/quite easy) vs “difficult” (quite difficult/very difficult);
8. View on implementation of label in United Kingdom—support for mandatory labelling (yes-all); support for voluntary labelling (yes-some); no support for labels (no-none);
9. View on helpfulness of label for food shopping—dichotomised as “helpful” (very helpful/quite helpful) vs “not helpful” (not very helpful/not at all helpful);
10. View on time to use label when shopping—quick enough/too long.

#### 2.5.3. Participant Characteristics/Covariates

Food shopping and eating habits were assessed by asking food-shopping responsibility, label use when shopping, influence of nutritional information on shopping, healthy-eating knowledge, healthy-eating interest, if currently trying to lose weight (see Supplementary Table S1 for questionnaire). Demographics and health-related questions included height and weight, pregnancy status, physical or mental health conditions that affect vision/learning, understanding or concentration/diet for 12 months and English as a second language. As panel members of NatCen, certain demographic information was already known (sex, age), and other key demographic information was only asked if it had been more than 6 months since last updated (income, educational level). Additional information on household composition (children under 16 years) was also collected.

### 2.6. Statistical Analysis 

The experiment was powered on primary outcomes, and so testing on secondary outcomes was limited. We limited formal statistical analyses to healthiest-product outcomes and speed-of-ranking outcomes, with a focus on the N-S-and-MTL comparison. For the healthiest-food-outcome analyses, we used the same three tests as for the primary analyses [18]: multilevel log-binomial regression analysis to compare the proportion of correct rankings between baseline and follow-up; log-binomial regression to compare the change in rankings from baseline to follow-up; and multiple linear regression analysis to compare the change in the global food score from baseline to follow-up. For the speed-of-ranking analyses, we log-transformed the data to normalize the distribution, and then used regression analysis to test the association between the time taken to rank products at follow-up, before back transforming for interpretation. For these four outcomes, we compared each FOPL group with the control, and additionally compared MTL and N-S.

All models were adjusted for the five stratification factors used for randomisation (year of recruitment to panel, sex, age, government-office region, and household income) and the following prespecified covariates: ethnicity, highest education level, household composition, food-shopping responsibility and current FOPL use. For product-level analyses, we excluded participants if they self-reported not buying or consuming that product in the last 12 months to ensure that a lack of familiarity with a product did not impact results (see Supplementary Table S3 for sensitivity analysis with no requirement to consume products). For global-food-score analysis, we only included participants who reported buying or consuming all five food products, and so a lack of familiarity with a product did not impact the results. For speed of ranking, we required participants to correctly rank the product under the label condition (see Supplementary Table S4 for sensitivity analysis with no requirement to be correct at follow-up). Additionally, for the speed-of-ranking outcome, we adjusted for device used, as mobile users were found to be faster (coded as mobile, desktop or both—as participants could leave and rejoin the survey; see Supplementary Table S2 for the proportion of participants and devices used).

Models were weighted to account for non-responses and to ensure findings were representative of the British adult population. Stata software (Release 16, StataCorp LLC., College Station, TX, USA) was used for all analyses, and a significance level of 5% was used [23]. Output for healthiest-product outcomes was presented as relative risk (RR) of linear regression coefficient, with 95% confidence intervals, and output for speed of ranking was presented as relative mean (RM) (i.e., the ratio of the mean outcome in one group relative to the mean outcome in another group). The associations between label perceptions and FOPL groups are presented descriptively. Descriptive findings for the global change score (see primary outcome paper for details [18]) are presented by equivalized income per month (more than GBP 2000; GBP 1251–2000; GBP 801–1250; GBP 800 or less).

## 3. Results

### 3.1. Participant Characteristics

NatCen invited 7218 panel members to participate, of whom 4863 accepted the invitation and were randomized to five experimental conditions: MTL (*n* = 968), N-S (*n* = 985), WL (*n* = 967), PC (*n* = 966) and control (*n* = 977). Complete data were available for 4530 participants and were included in these analyses. The participant characteristics for the food and shopping variables are presented in Table 1 (see Supplementary Table S4 for participant by characteristics by experimental group, and primary analyses for full baseline characteristics). The analysis sample is broadly representative of the British population and of the full NatCen sample [24]. There was a high proportion of participants who reported being very or quite interested in healthy eating (94%) and having some or a lot of knowledge about healthy eating (86%). Nearly half of the sample reported that they were currently trying to lose weight (47%).

### 3.2. Healthiest-Product Outcome 

**Figure 1:**
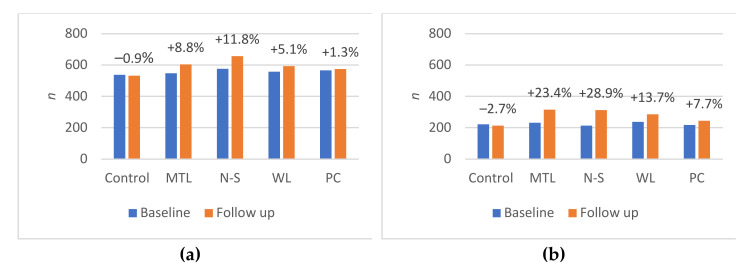

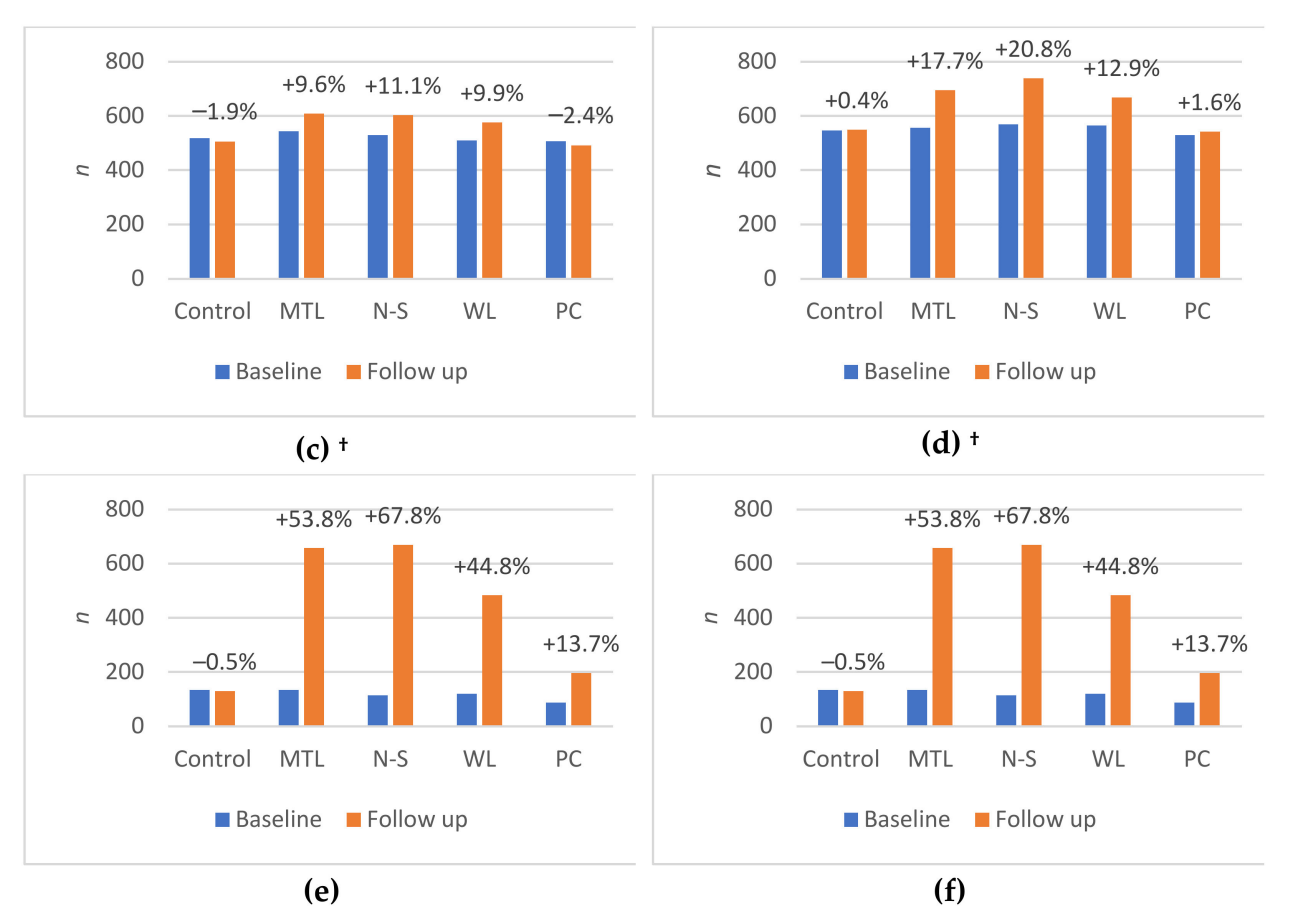
Number of participants who correctly ranked the healthiest product at baseline and follow-up, by FOPL group and product category: (**a**) pizza; (**b**) drink; (**c**) cake; (**d**) crisps; (**e**) yoghurt; (**f**) cereal. ^†^ Cake and crisps categories had no products qualify for Positive Choice tick. MTL: Multiple Traffic Lights; N-S: Nutri-Score; WL: Warning Label; PC: Positive Choice tick; FOPL: Front of pack label.

Figure 1 shows the percentage of participants who correctly ranked the healthiest product at baseline and follow-up, and the percent change by experiment group and product category (see Table S5 for the number and proportion of participants correctly ranking at baseline and follow-up). The associations between the experimental group and the correct ranking of the healthiest product at follow-up, adjusted for covariates, are shown below in Table 2. The probability of participants correctly ranking the healthiest product at follow-up was significantly greater across all product categories in MTL, N-S and WL, compared to the control. N-S had the highest RR of correctly ranking the healthiest product, followed by MTL and WL. The PC group had mixed results, with significant differences for drink, yoghurt and cereal, but not for pizza, crisps or cake (no products in the cake and crisps categories were eligible for a PC). There were no significant differences between MTL and N-S in the direct comparison. We found that the proportion of participants correctly ranking the healthiest product at baseline was similar across the FOPL conditions.

The associations between FOPLs and the improved-change score, adjusted for covariates, are shown in Table 3. The probability of participants improving their score from baseline to follow-up was significantly greater across all product categories in MTL, N-S and WL, compared to the control. N-S led to the greatest probability of improved scores, followed by MTL and WL. PC had mixed results, with a greater probability of improved scores for drinks, yoghurt and cereal, but not pizza, cake or crisps. The comparison between N-S and MTL showed no significant difference.

The associations between an improved healthiest-product global food score and the FOPL groups, adjusted for covariates, are shown in Table 4. We found that all of the FOPL groups were associated with a significant increase in the global food score for the healthiest product, compared to the control. A comparison between N-S and MTL showed that N-S led to a greater improvement in the healthiest-product global food score.

### 3.3. Speed of Ranking

**Figure 2:**
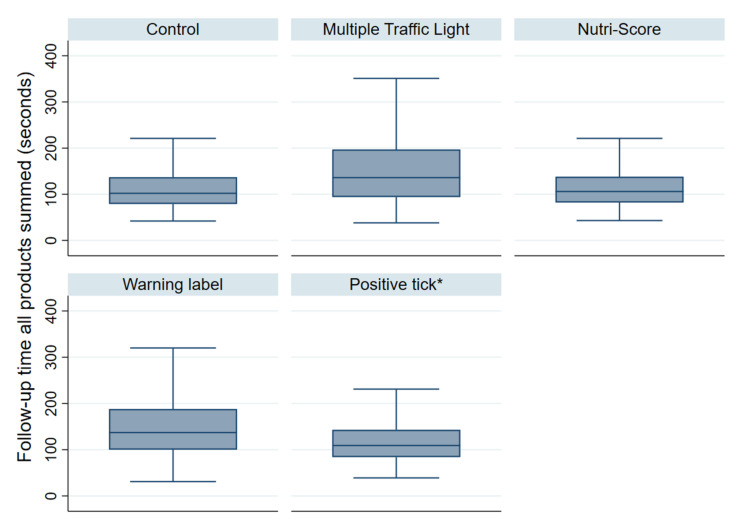
Median and interquartile ranges of ranking times (seconds) of summed follow-up rankings by experimental condition, excluding participants who did not have full covariate information and outliers. * Cake and crisps categories had no products qualify for Positive Choice tick; outliers not presented in the graph.

The median and interquartile ranges of the time to complete the baseline and follow-up rankings in seconds, by experimental group and product category, are shown in Appendix A Table A1. The baseline median ranking times were similar within product categories and between groups. The follow-up rankings were faster compared to the baseline rankings, for all categories and experimental groups. Overall, there was variation between the product categories at baseline (cake was the quickest, yoghurt and cereal were the longest), but there was little variation between the product categories at follow-up. In a comparison between N-S and MTL, N-S was fastest across all categories. The median ranking times and the interquartile ranges, in seconds, of the summed follow-up ranking times (seconds) by experimental condition are shown in Figure 2.

The associations between the time to complete the rankings of each product and the FOPL conditions, adjusted for covariates, are shown in Table 5, with a higher RM indicating that the time to complete the ranking was slower (i.e., worse) (see Supplementary Table S6 for sensitivity analysis without the requirement for being correct at follow-up). By FOPL group, for pizza, we found that the time to complete the ranking significantly increased by 25% for MTL, and by 28% for WL, compared to the no-label control; there were no significant differences for N-S or PC compared to the no-label control. The pattern was broadly consistent across the product categories. A comparison between N-S and MTL showed that the time to complete the ranking decreased by 15–22% for the participants in the N-S group compared to the MTL group, across all product categories (all *p* < 0.001).

### 3.4. Descriptive Analyses of Perceptions

Descriptive analyses of the label perceptions by FOPL group are shown in Table 6. We did not formally test the differences between the labels. These questions were specific to the label conditions that the participants were randomised to and were therefore not applicable to the control group, as they saw no labels. Overall, the perceptions of the labels differed greatly between the FOPL groups. The majority of participants favourably viewed labels and supported mandatory labelling in the United Kingdom. The N-S and MTL groups had the highest proportions of participants perceive labels favourably. N-S had the highest proportion of participants who reported: seeing the label; using the label for all ranking tasks; that the label was easy to understand; and that the label was quick enough to use. Most commonly, MTL followed. MTL had the highest proportion of participants who reported perceiving the label to be helpful in making purchasing decisions and who supported having labels on all food and drink products in the United Kingdom.

### 3.5. Descriptive Analysis of Ranking Outcome by Income

Descriptive analyses of the global food score by experimental group and participant income are shown in Table 7, with the higher global food score indicating greater improvement in ranking (see Supplementary Table S7 for sensitivity analysis without requirement to consume all food products). Between N-S and MTL, N-S appeared to be more stable at a high level and showed fewer differences in the global food score across income groups, compared to MTL. The experiment was not powered to formally test these differences.

## 4. Discussion

In this online randomized controlled experiment on a representative British sample, we found that N-S and MTL performed the best across a range of outcomes, including the correct ranking of the healthiest product and the speed of the ranking. The healthiest-product analysis found that N-S, MTL and WL performed the best, and PC performed the worst in the ranking tasks. In terms of timing, the analysis showed that participants who used N-S ranked significantly faster than those who used MTL, and the descriptive analysis of the label perceptions showed that FOPLs were perceived favourably overall, and especially N-S and MTL. This is consistent with previously published data on the primary outcomes (i.e., that N-S, followed by MTL, led to significant improvements in the ability of participants to correctly rank products according to healthiness).

Our findings are broadly consistent with previous research. The results from experimental ranking studies have shown that N-S performs the best for ranking tasks [10,11], and a meta-analysis found that summary indicator labels are the most effective at helping consumers identify the healthiest products [16]. In the current study, we conducted the healthiest-product analysis to supplement the primary analysis of ranking all three correctly in order to allow for a fairer comparison for PC (except cake and crisps, which did not qualify). Since PC is a binary label, it cannot differentiate between three products. Despite this, PC was still found to be the worst-performing label. For the time needed to use labels, no studies have compared N-S and MTL, to the best of our knowledge, but experimental studies conducted in Uruguay and Mexico found that MTL and WL were faster compared to the Guideline Daily Amounts [25,26]. We found that, in a large representative British sample, N-S was significantly faster to use than MTL for the follow-up ranking task, and, crucially, it also led to the highest proportion of correct rankings in the primary analyses, compared to the other experimental groups. This is interesting as the MTL label is currently endorsed and used on a voluntary basis in the United Kingdom, and it is therefore likely to be familiar to most participants. N-S provides an overall summary of the product healthiness, compared to MTL, which contains more information, including nutrient-specific information. Experimental studies that tested the perceptions of FOPLs (including MTL and N-S) following use in the ranking tasks found that the respondents viewed the labels favourably, in general, and that they strongly supported mandatory labelling across all label conditions [27,28,29]. They found that the participants who viewed MTL responded most favourably across 12 countries [27], but later replications in Belgium and the Netherlands found no difference [28,29]. A large cross-sectional study in France found N-S was most favourably perceived by the participants, who also rated MTL, a reference intake label and a simplified nutrition-labelling system, including the quickest to process [30].

Time has been identified as a factor that impacts upon actual label use, and so our finding that N-S is the fastest to use while also leading to the highest proportion of correctly ranked products suggests that N-S may be a more effective label than the other labels that we tested [8,9,31,32]. The ranking tasks in this study are not representative of how long the tested labels would take to use in real decision-making scenarios with additional factors, such as costs, preferences, and product claims, but we believe that these factors are likely to be broadly consistent, regardless of the FOPL used. Indeed, research indicates that people spend less time making purchasing decisions than the median time shown to complete the ranking tasks, with observational studies in the United Kingdom and Uruguay showing that consumers spend, on average, 22–28 s choosing a product, and only 7 s from picking up a product to putting in a basket [6,33].

As was previously stated, none of the labels were explained to the participants, and we assumed that prior exposure to labels other than MTL (in voluntary use in England) was low; therefore, we expected a low subjective understanding of these labels. Both N-S and PC are abstract labels that may need some explanation, which is contrary to MTL and WL, which are self-explanatory. Because of this, the positive perceptions of N-S and MTL indicate that both summary indicator and nutrient-specific labels may be liked by British consumers. MTL is the FOPL that is currently used in the United Kingdom on a voluntary basis, and so the understanding and use of this label is assumed to be higher compared to the other labels. Understanding and familiarity combined may have negatively impacted on the confidence in using the other labels, compared to MTL [7,34].

The secondary outcomes of timing and perception that are presented here relate to the actual use of FOPLs, as opposed to the ability of FOPLs to improve the understanding of product healthiness. For FOPLs to be effective, they not only need to easily communicate nutritional information about the product, but they also need to be useable, and especially for people with lower education. This is vital, as a key aim for policymakers is to reduce, and not widen, inequalities. The descriptive results indicate the N-S may work better across all income groups, as the global-food-score results were more stable, compared to MTL and WL. We found a similar result when examining the proportion of correct/incorrect by education level [18].

Despite being a high-quality experimental study, there were limitations of the secondary analyses. The study was powered on the primary aims, and so we limited the statistical testing of the secondary aims. The healthiest-product analysis aimed to deal with the limitations of the PC label, as it does not provide sufficient information to rank products because it is a binary label. A further limitation of the PC label is that it did not qualify for use in two of the food categories (crisps, cakes), which is reflected in the responses of the participants in the PC group who reported seeing the label (lower compared to the other FOPL groups). This is reflective of the real-world application and limitations of the PC label, as many products would not qualify and, therefore, would not have a label or provide any information to consumers. Data from the software was used for the timing outcomes, and the participants were given the following instructions: “we would like to know how long it takes for you to rank the different foods, so please click ‘next’ as soon as you have completed each task.” These directions were provided not only to make the participants aware that we would collect this data, but also so as not to rush them, as timing was not the primary outcome and, therefore, care needs to be taken with the interpretation of the findings. The participants only answered perception questions about the FOPL that they used, and, therefore, we cannot compare preferences between labels. This was not only partly to reduce the time burden on the participants, but it also meant that the responses to these questions were based on the actual use of the label. The sample was representative of adults in Great Britain and was broadly comparable to the full NatCen panel and to the British population, including the percentage who reported wanting to lose weight [24,35,36].

## 5. Conclusions

The results from this randomised controlled experiment show that FOPLs can be used quickly and effectively to improve the understanding of product healthiness, and that they are perceived favourably in a large representative British sample. The findings were consistent with the primary report, and they show that N-S performed the best across the outcomes, and that it was closely followed by MTL; all FOPLs were better than the no-label control. The N-S label was also the quickest to use correctly. The FOPLs were perceived favourably overall, and especially MTL and N-S, with strong support for mandatory labelling on all products in the United Kingdom. The findings of this experiment extend the evidence and provide support for FOPLs in the United Kingdom, which can inform future policy decisions.

## Figures and Tables

**Table 1:** Baseline characteristics—food and shopping variables.

	*n* (%)
**Shopping responsibility**	
Yes—some or all	4340 (96)
No—someone else does	190 (4)
**Current label use**	
Very often	945 (21)
Quite often	1546 (34)
Occasionally	1318 (29)
Rarely	559 (12)
Never	162 (4)
**Reported consuming or buying product in past 12 months**	
Pizza	3361 (75)
Drink	1630 (36)
Cake	3163 (70)
Crisps	3723 (83)
Yoghurt	3779 (84)
Breakfast cereal	3802 (84)
**Currently trying to lose weight**	
Yes	2125 (47)
No	2240 (49)
Prefer not to say	165 (4)
**Interested in healthy eating**	
Very interested	1894 (42)
Quite interested	2332 (51)
Not very interested	280 (6)
Not at all interested	24 (1)
**Knowledge of healthy eating**	
A lot of knowledge	1275 (28)
Some knowledge	2629 (58)
A little knowledge	602 (13)
No knowledge	23 (1)

**Table 2:** Multilevel log-binomial regression results—healthiest product ranked correctly (yes/no) at follow-up (adjusted for baseline rank), compared to control by FOPL group (adjusted for design effects and covariates).

	MTL vs Control RR (95% CI)	N-S vs Control RR (95% CI)	WL vs Control RR (95% CI)	PC vs Control RR (95% CI)	N-S vs MTL RR (95% CI)
**Pizza**	1.12	1.2	1.09	1.03	1.07
	(1.04, 1.20)	(1.12, 1.28)	(1.03, 1.16)	(0.97, 1.09)	(0.99, 1.15)
	*p =* 0.002	*p* < 0.001	*p* = 0.005	*p* = 0.29	*p* = 0.10
**Drink**	1.42	1.57	1.2	1.22	1.11
	(1.26, 1.59)	(1.39, 1.78)	(1.08, 1.34)	(1.10, 1.34)	(0.97, 1.26)
	*p <* 0.001	*p <* 0.001	*p* = 0.001	*p <* 0.001	*p* = 0.12
**Cake**	1.2	1.22	1.19	1.02 ^†^	1.02
	(1.12, 1.29)	(1.14, 1.31)	(1.12, 1.26)	(0.97, 1.07)	(0.94, 1.10)
	*p <* 0.001	*p <* 0.001	*p <* 0.001	*p* = 0.45	*p* = 0.63
**Crisps**	1.26	1.31	1.2	1.04 ^†^	1.04
	(1.17, 1.35)	(1.22, 1.40)	(1.12, 1.28)	(0.97, 1.10)	(0.96, 1.13)
	*p <* 0.001	*p <* 0.001	*p <* 0.001	*p* = 0.26	*p* = 0.37
**Yoghurt**	4.89	5.64	3.81	2.5	1.15
	(3.78, 6.33)	(4.22, 7.54)	(2.83, 5.12)	(1.89, 3.30)	(0.85, 1.57)
	*p <* 0.001	*p <* 0.001	*p <* 0.001	*p <* 0.001	*p* = 0.37
**Cereal**	1.95	1.98	1.76	1.18	1.02
	(1.69, 2.24)	(1.71, 2.30)	(1.49, 2.06)	(1.03, 1.35)	(0.88, 1.17)
	*p <* 0.001	*p <* 0.001	*p <* 0.001	*p* = 0.018	*p* = 0.84

^†^ Cake and crisps categories had no products qualify for Positive Choice tick; all analyses were adjusted for baseline ranking (correct/incorrect), stratification factors (year of recruitment to panel, sex, age, government-office region, household income), and the following prespecified covariates: ethnicity, highest education level, household composition, food-shopping responsibility, and current FOPL use. MTL: Multiple Traffic Lights; N-S: Nutri-Score; WL: Warning Label; PC: Positive Choice tick; RR: relative risk; CI: confidence interval.

**Table 3:** Log-binomial regression results—relative risk that ranking of healthiest product improved (follow-up vs baseline) between FOPL group and control (adjusted for design factors and covariates).

	MTL vs Control RR (95% CI)	N-S vs Control RR (95% CI)	WL vs Control RR (95% CI)	PC vs Control RR (95% CI)	N-S vs MTL RR (95% CI)
**Pizza**	2.91	3.29	2.57	1.35	1.13
	(1.81, 4.68)	(2.05, 5.27)	(1.60, 4.15)	(0.81, 2.26)	(0.79, 1.61)
	*p* < 0.001	*p* < 0.001	*p* < 0.001	*p* = 0.24	*p* = 0.50
**Drink**	8.04	10.68	5.06	3.98	1.33
	(3.83, 16.86)	(5.16, 22.09)	(2.37, 10.78)	(1.74, 9.09)	(0.95, 1.85)
	*p* < 0.001	*p* < 0.001	*p* < 0.001	*p* = 0.001	*p* = 0.09
**Cake**	8.59	8.98	7.57	1.07 ^†^	1.05
	(4.45, 16.60)	(4.70, 17.16)	(3.94, 14.57)	(0.41, 2.76)	(0.71, 1.54)
	*p* < 0.001	*p* < 0.001	*p* < 0.001	*p* = 0.89	*p* = 0.82
**Crisps**	4.86	5.65	3.73	1.62 ^†^	1.16
	(3.05, 7.72)	(3.60, 8.97)	(2.30, 6.03)	(0.87, 3.00)	(0.91, 1.49)
	*p* < 0.001	*p* < 0.001	*p* < 0.001	*p* = 0.13	*p* = 0.23
**Yoghurt**	18.13	18.5	13.07	4.57	1.02
	(10.78, 30.49)	(10.99, 31.15)	(7.72, 22.11)	(2.62, 7.99)	(0.93, 1.12)
	*p* < 0.001	*p* < 0.001	*p* < 0.001	*p* < 0.001	*p* = 0.68
**Cereal**	5.2	5.78	4.73	1.95	1.11
	(3.50, 7.73)	(3.90, 8.56)	(3.17, 7.08)	(1.26, 3.01)	(0.96, 1.29)
	*p* < 0.001	*p* < 0.001	*p* < 0.001	*p* = 0.003	*p* = 0.17

^†^ Cake and crisps categories had no products qualify for Positive Choice tick; all analyses were adjusted for the five stratification factors (year of recruitment to panel, sex, age, government-office region, household income) and the following prespecified covariates: ethnicity, highest education level, household composition, food shopping. MTL: Multiple Traffic Lights; N-S: Nutri-Score; WL: Warning Label; PC: Positive Choice tick; RR: relative risk; CI: confidence interval.

**Table 4:** Multiple regression analysis results—association of global food score between FOPL group and control (adjusted for design factors and covariates).

	MTL vs Control RR (95% CI)	N-S vs Control RR (95% CI)	WL vs Control RR (95% CI)	PC vs Control RR (95% CI)	N-S vs MTL RR (95% CI)
**Score (−5, +5)**	1.5	1.8	1.2	0.3	0.3
**Regression**	(1.3, 1.6)	(1.6, 1.9)	(1.0, 1.3)	(0.2, 0.5)	(0.2, 0.5)
**(coefficients)**	*p <* 0.001	*p <* 0.001	*p <* 0.001	*p <* 0.001	*p <* 0.001

Global food score was an aggregated score of correct ranking of the healthiest product in the five food products, with a range from −5 to +5 (− indicates worsening and + indicates improvement); all analyses adjusted for stratification factors (year of recruitment to panel, sex, age, government-office region, household income) and covariates: ethnicity, highest education level, household composition, food-shopping responsibility, current FOPL use. MTL: Multiple Traffic Lights; N-S: Nutri-Score; WL: Warning Label; PC: Positive Choice tick; RR: relative risk; CI: confidence interval.

**Table 5:** Multiple regression analysis results—association between time taken to rank products at follow-up and FOPL group compared to control (adjusted for baseline ranking time, device used, design factors and covariates).

	MTL vs Control RM (95% CI)	N-S vs Control RM (95% CI)	WL vs Control RM (95% CI)	PC vs Control RM (95% CI)	N-S vs MTL RM (95% CI)
**Pizza**	1.25	1	1.28	1.05	0.8
	(1.17, 1.33)	(0.94, 1.06)	(1.20, 1.37)	(0.98, 1.12)	(0.75, 0.85)
	*p* < 0.001	*p* = 0.96	*p* < 0.001	*p* = 0.16	P < 0.001
**Drink**	1.28	1.08	1.28	1.1	0.85
	(1.18, 1.38)	(1.00, 1.17)	(1.18, 1.39)	(1.01, 1.19)	(0.79, 0.91)
	*p* < 0.001	*p* = 0.056	*p* < 0.001	*p* = 0.035	*p* < 0·001
**Cake**	1.43	1.18	1.61	1.09	0.82
	(1.35, 1.53)	(1.10, 1.26)	(1.51, 1.72)	(1.02, 1.17)	(0.78, 0.86)
	*p* < 0.001	*p* < 0.001	*p* < 0.001	*p* = 0.01	*p* < 0·001
**Crisps**	1.39	1.13	1.3	1.12	0.82
	(1.31, 1.46)	(1.07, 1.19)	(1.23, 1.38)	(1.05, 1.19)	(0.78, 0.86)
	*p* < 0.001	*p* < 0.001	*p* < 0.001	*p* < 0.001	*p* < 0·001
**Yoghurt**	1.4	1.1	1.44	1.16	0.78
	(1.26, 1.56)	(0.99, 1.22)	(1.29, 1.60)	(1.01, 1.33)	(0.74, 0.83)
	*p* < 0.001	*p* = 0.08	*p* < 0.001	*p* = 0.10	*p* < 0·001
**Cereal**	1.31	1.02	1.41	1.11	0.78
	(1.22, 1.41)	(0.95, 1.10)	(1.31, 1.52)	(1.02, 1.21)	(0.74, 0.82)
	*p* < 0.001	*p* = 0.08	*p* < 0.001	*p* = 0.03	*p* < 0·001

All analyses were adjusted for the five stratification factors (year of recruitment to panel, sex, age, government-office region, household income) and the following prespecified covariates: ethnicity, highest education level, household composition, food-shopping responsibility, current FOPL use, baseline ranking time and device used. Participants needed to have complete covariate information and buy/eat cereal/pizza to be included, and they needed to correctly rank the products. To deal with outliers, the data were log-transformed for analyses and then back transformed for interpretation. MTL: Multiple Traffic Lights; N-S: Nutri-Score; WL: Warning Label; PC: Positive Choice tick; RM: relative mean; CI: confidence interval.

**Table 6:** Proportions of participant FOPL perception outcomes, by FOPL group.

	MTL (*n* = 907) *n* (%)	N-S (*n* = 924) *n* (%)	WL (*n* = 895) *n* (%)	PC (*n* = 891) *n* (%)
**Saw label**				
Yes	697 (77)	807 (87)	698 (78)	490 (55)
No/not sure	210 (23)	117 (13)	197 (22)	401 (45)
**Used label**				
All	627 (69)	697 (75)	479 (54)	88 (10)
Some	51 (6)	85 (9)	190 (21)	214 (24)
Did not use	19 (2)	25 (3)	29 (3)	186 (21)
Not applicable	210 (23)	117 (13)	197 (22)	401 (45)
**Understanding labels**				
Easy	669 (74)	717 (78)	601 (67)	273 (31)
Difficult	28 (3)	89 (10)	97 (11)	217 (24)
Not applicable	210 (23)	117 (13) ^^^	197 (22)	401 (45)
**Label helpfulness**				
Helpful	885 (98)	845 (91)	822 (92)	660 (74)
Not helpful	22 (2)	79 (9)	73 (8)	230 (26) ^^^
**Time to use label**				
Quick enough	846 (93)	902 (98)	816 (91)	821 (92)
Too long	60 (7)	21 (2)	77 (9)	67 (8)
**Labels on products in UK**				
Yes-all	813 (90)	718 (78)	667 (75)	451 (51)
Yes-some	81 (9)	150 (16)	174 (19)	289 (32)
No-none	13 (1)	56 (6)	54 (6)	148 (17) *

* 3 do not know responses; ^ 1 does not know response. Control group not included as they were not asked these questions. Participants needed to have full covariate information to be included. Helpful dichotomised as helpful (very helpful, quite helpful) vs not helpful (not very helpful, not at all helpful). MTL: Multiple Traffic Lights; N-S: Nutri-Score; WL: Warning Label; PC: Positive Choice tick.

**Table 7:** Mean global food scores and standard deviations by experimental group and equivalised household income per month.

	Control (*n* = 384) Mean (SD)	MTL (*n* = 393) Mean (SD)	N-S (*n* = 395) Mean (SD)	WL (*n* = 413) Mean (SD)	PC (*n* = 391) Mean (SD)	Overall (*n* = 1976) Mean (SD)
**Equivalised income per month**						
More than GBP 2000	0.0 (0.6)	2.1 (1.2)	2.0 (1.1)	1.6 (1.4)	0.2 (0.9)	1.2 (1.4)
GBP 1251–2000	−0.0 (0.8)	2.2 (1.3)	2.1 (1.3)	1.4 (1.4)	−0.0 (0.8)	1.2 (1.5)
GBP 801–1250	0.0 (0.8)	1.9 (1.2)	2.0 (1.2)	1.5 (1.4)	0.2 (0.9)	1.1 (1.4)
GBP 800 or less	0.1 (0.7)	1.3 (1.7)	2.2 (1.1)	1.1 (1.6)	0.3 (1.1)	1.0 (1.5)

Global food score is an aggregated score of correct ranking in the five food products, with a range from −5 to +5 (− indicates worsening and + indicates improvement). Participants required to buy/eat each food product in the last 12 months and to have full covariate information to be included. MTL: Multiple Traffic Lights; N-S: Nutri-Score; WL: Warning Label; PC: Positive Choice tick; SD: standard deviation.

## Data Availability

The data presented in this article are available upon reasonable request from the corresponding author.

## Supplementary Materials

The following supporting information can be downloaded at: https://www.mdpi.com/article/10.3390/nu14112188/s1, Table S1: Full questionnaire; Table S2: Device usage by age category; Table S3: Summary of baseline and follow-up ranking times (seconds), by product category, overall and FOPL group (no requirement to consume product to be included in analysis); Table S4: Individual characteristics of the analysis sample, by experimental group; Table S5: Summary of participants who correctly ranked the healthiest product at baseline and follow-up, by FOPL group and product category; Table S6: Multiple regression analysis results—association between time taken to rank products at follow-up and FOPL group compared to control—without requirement for being correct (adjusted for baseline ranking time, device used, design factors and covariates); Table S7: Mean global food score and standard deviations by experimental group and equivalised household income per month—without requirement of buying/eating all food products.

## Appendix A

**Table A1:** Summary of baseline and follow-up ranking times (seconds) by product category, overall and experimental group.

	Control (*n* = 913 *) Median (IQR)		MTL (*n* = 907 *) Median (IQR)		N-S (*n* = 924 *) Median (IQR)		WL (*n* = 895 *) Median (IQR)		PC (*n* = 891 *) Median (IQR)		Overall (*n* = 4530 *) Median (IQR)	
	**Baseline**	**Follow-up**	**Baseline**	**Follow-up**	**Baseline**	**Follow-up**	**Baseline**	**Follow-up**	**Baseline**	**Follow-up**	**Baseline**	**Follow-up**
**Pizza**	32	16	34	19	34	16	33	21	33	16	33	17
	(22–47)	(11–23)	(24–48)	(13–31)	(25–52)	(12–23)	(23–48)	(14–30)	(23–47)	(12–24)	(24–48)	(12–26)
**Drink**	30	14	30	18	31	15	29	18	30	15	30	16
	(20–41)	(9–21)	(21–47)	(13–28)	(23–45)	(11–21)	(21–44)	(13–25)	(22–43)	(11–20)	(22–44)	(11–23)
**Cake**	25	14	27	19	26	15	26	22	25	15	26	16
	(18–37)	(9–20)	(19–38)	(13–31)	(18–39)	(12–22)	(18–37)	(14–34)	(18–37)	(10–23)	(18–38)	(11–26)
**Crisps**	29	15	29	20	30	16	29	20	29	17	29	18
	(21–41)	(10–21)	(21–40)	(14–30)	(22–41)	(12–24)	(21–41)	(14–28)	(21–40)	(12–24)	(21–41)	(12–26)
**Yoghurt**	36	16	36	21	35	16	36	21	35	17	36	18
	(25–53)	(11–24)	(25–53)	(14–32)	(25–52)	(12–23)	(24–51)	(14–30)	(23–50)	(12–25)	(24–52)	(12–27)
**Cereal**	35	17	36	21	36	17	36	23	34	17	36	19
	(24–53)	(12–25)	(25–55)	(15–33)	(25–52)	(13–23)	(25–53)	(16–33)	(24–50)	(13–27)	(24–52)	(13–28)

* Sample size differed as participants needed to have complete covariate information and buy/eat each product to be included in each product analysis. IQR: interquartile range; MTL: Multiple Traffic Lights; N-S: Nutri-Score; WL: Warning Label; PC: Positive Choice tick; RR: relative risk; CI: confidence interval.
