# Supplementary material for: Secondary Outcomes of a Front-of-Pack-Labelling Randomised Controlled Experiment in a Representative British Sample: Understanding, Ranking Speed and Perceptions
Source: Nutrients. 2022 May 24;14(11):2188. doi: 10.3390/nu14112188 (PMC9182518; doi:10.3390/nu14112188)
Supplement: Supplementary file 1 [file nutrients-14-02188-s001.zip › nutrients-1722426-supplementary.pdf]

Supplementary file

Table S1. Full questionnaire

1.1 Baseline ranking

Our first set of questions will ask you to rank different foods on how healthy you think they are. We would like to know how long it takes for you to rank the different foods, so please click 'next' as soon as you have completed each task.

Please rank them from most healthy to least healthy

Baseline ranking task

Below are three pictures of different types of pizza. Please select how healthy each pizza is compared to the other 2 pizzas – most healthy, least healthy or in between.

Please select one answer for each image.

[Images of the 3 pizzas presented here with answer boxes below each image: 1 – Most healthy, 2 – In between, 3 – Least healthy]

- If participants ranked two products the same this message appeared: You have ranked two foods the same. If you are not sure what order to put the foods in, please give your best estimate.

Enough information question

Do you feel you had enough information to rank the pizzas from most healthy to least healthy?"

1. Yes
2. No

[Ranking task and enough information question repeated for the 5 other product categories]

Confidence question

Thinking about **all of** the rankings you have just done…

How many do you think you ranked in the correct order – from most healthy to least healthy?

1. All of them
2. Most of them
3. Some of them
4. None of them

1.2 Experimental ranking

We will now present you with another set of pictures of the same food items. Remember, we would like to know how long it takes for you to rank the different foods, so please click 'next' as soon as you have completed each task.

Please rank them from most healthy to least healthy.

Experimental ranking task

Below are three pictures of different types of pizza. Please select how healthy each pizza is compared to the other 2 pizzas – most healthy, least healthy or in between.

Please select one answer for each image.

[Images of the 3 pizzas with allocated FOPL presented here with answer boxes below each image: 1 – Most healthy, 2 – In between, 3 – Least healthy]

- If participants had two products ranked the same this message appeared: You have ranked two foods the same. If you are not sure what order to put the foods in, please give your best estimate.

Enough information question

Do you feel you had enough information to rank the pizzas from most healthy to least healthy?"

1. Yes
2. No

[Ranking task and enough information question repeated for the 5 other product categories]

Confidence question

Thinking about **the second set** of rankings you have just done…

How many do you think you ranked in the correct order – from most healthy to least healthy?

1. All of them
2. Most of them
3. Some of them
4. None of them
5. Don't know

POST-RANKING QUESTIONS

1.3 Labels (excluding control)
Thinking about the second set of pictures of food we showed you, did you see any labels that looked like the following?
[shown image of their allocated FOPL condition]

1. Yes
2. No
3. Not sure

And did you those labels to help you rank how healthy the different foods and drinks are?

1. I used the labels to help me rank **all** of the foods
2. I used the labels to help me rank **some** of the foods
3. I did not use the labels to help me rank the foods

And how easy or difficult did you find it to **understand** these labels?

1. Very easy
2. Quite easy
3. Quite difficult
4. Very difficult

Would you like these labels to be put on food and drink packaging in the UK?

1. Yes - all
2. Yes – some
3. No - none

How helpful do you think you would find these labels in choosing what to buy?

1. Very helpful
2. Quite helpful
3. Not very helpful
4. Not at all helpful

Which of the following best represents your views on how long it takes to use these labels?

1. These labels would take too long to use when buying food and drink
2. These labels would be quick enough to use when buying food and drink

1.4 Food habits
Our next set of questions are about your current food shopping and eating habits
Do you ever do any food shopping, either for yourself or someone else?

1. Yes - I do some food shopping
2. No - someone else does food shopping for me

How often do you read the nutritional information on the front of packaging when buying food or drink?

1. Very often
2. Quite often
3. Occasionally
4. Rarely
5. Never

And how often does the nutritional information on food packaging influence what you buy?

1. Very often
2. Quite often
3. Occasionally
4. Rarely
5. Never

How much **knowledge** would you say you have about healthy eating?

1. A lot of knowledge
2. Some knowledge
3. A little knowledge

4. No knowledge

How **interested** would you say you are in healthy eating?

1. Very interested
2. Quite interested
3. Not very interested
4. Not at all interested

Are you currently trying to lose weight?

1. Yes
2. No
3. Prefer not to say

Thinking about the past 12 months, which of the following types of food or drink have you bought or consumed?
Please select all that apply

1. Pizza
2. Hot chocolate
3. Cake
4. Crisps
5. Yoghurt
6. Breakfast cereal
7. None of these

1.5 Background questions
Our final set of questions is to help us with our analysis.
We would like to know how to tall you are.
Would you prefer to tell us your height in metres and centimetres or feet and inches?

1. Metres and centimetres
2. Feet and inches
3. I'd prefer not to give my height

What is your height without shoes?
If you are unsure, please give your best estimate
[Textbox response] (range 0…2 metres, 0…99 centimetres OR 0…40 stones, 0…13 pounds)

We would like to know how much you weigh.
Would you prefer to tell us your weight in kilograms or stones and pounds?

1. Kilograms
2. Stones and pounds
3. I'd prefer not to give my weight

How much do you weigh without clothes and shoes?
If you are unsure, please give your best estimate
[Textbox response] (range 0…250 kilograms OR 0…7 feet, 0…11 inches)

Are you currently pregnant?

1. Yes
2. No
3. Prefer not to say

Do you have any physical or mental health conditions or illnesses lasting or expected to last for 12 months or more which affect **your vision** (for example blindness, or partial sight)?

1. Yes
2. No

Do you have any physical or mental health conditions or illnesses lasting or expected to last for 12 months or more which affect **your learning, understanding or concentration**?

1. Yes
2. No

Do you have any physical or mental health conditions or illnesses lasting or expected to last for 12 months or more which affect **what you eat**?<br>1. Yes<br>2. No
What is/are the physical or mental health condition(s) or illness(es) lasting or expected to last for 12 months or more which affect what you eat?<br>**[Textbox response]**
Has a doctor or other health professional ever told you that you have any of these conditions?<br>Please select all that apply.<br>1. Coronary heart disease<br>2. Angina<br>3. Heart attack or myocardial infarction<br>4. Type 1 Diabetes<br>5. Type 2 Diabetes<br>6. High blood pressure/hypertension<br>7. None of these
Is English your first language?<br>1. Yes<br>2. No

Table S2. Device usage by age category

	**Computer**	**Mobile**	**Both**	**Total**
	n (%)	*n* (%)	*n* (%)	*n*
**18-29**	74 (27)	203 (73)	2 (1)	279
**30-39**	146 (23)	492 (77)	4 (1)	642
**40-49**	272 (31)	603 (68)	6 (1)	881
**50-59**	398 (41)	558 (58)	13 (1)	969
**60-69**	476 (51)	446 (48)	11 (1)	933
**70+**	498 (61)	314 (38)	11 (1)	823

Table S3. Summary of baseline and follow-up ranking times (seconds), by product category, overall and FOPL group (with no requirement to consume product to be included in analysis)

	**Control (*n*=913) Median (IQR)**		**MTL (*n*=907) Median (IQR)**		**N-S (*n*=924) Median (IQR)**		**WL (*n*=895) Median (IQR)**		**PC (*n*=891) Median (IQR)**		**Overall (*n*=4530) Median (IQR)**	
	**Baseline**	**Follow-up**	**Baseline**	**Follow-up**	**Baseline**	**Follow-up**	**Baseline**	**Follow-up**	**Baseline**	**Follow-up**	**Baseline**	**Follow-up**
**Pizza**	34 (24-50)	17 (12-25)	35 (24-51)	20 (14-33)	34 (26-52)	16 (12-24)	34 (24-50)	22 (15-33)	35 (24-49)	17 (12-26)	35 (25-50)	18 (13-28)
**Drink**	33 (23-47)	15 (11-22)	34 (23-49)	20 (14-31)	33 (24-47)	16 (12-23)	32 (22-48)	19 (13-27)	32 (23-47)	16 (11-23)	33 (23-48)	17 (12-25)
**Cake**	25 (18-38)	14 (9-20)	27 (19-38)	19 (13-32)	27 (19-40)	16 (12-23)	27 (18-38)	22 (14-35)	25 (18-37)	15 (10-23)	26 (18-38)	17 (12-26)
**Crisps**	30 (22-42)	15 (11-22)	29 (22-41)	20 (14-32)	30 (22-42)	17 (12-24)	30 (22-43)	21 (15-29)	29 (21-41)	18 (12-25)	30 (22-42)	18 (13-26)
**Yoghurt**	36 (25-53)	16 (11-24)	36 (25-52)	21 (14-32)	36 (25-53)	16 (12-23)	36 (25-52)	21 (15-30)	35 (23-51)	17 (12-25)	36 (25-52)	18 (12-27)
**Cereal**	35 (24-52)	17 (12-25)	36 (25-54)	21 (14-33)	36 (25-52)	17 (16-32)	36 (25-52)	23 (16-32)	34 (24-50)	18 (12-27)	36 (24-52)	19 (13-28)

IQR: interquartile range; MTL: Multiple Traffic Lights; N-S: Nutri-Score; WL: Warning label; PC: Positive Choice tick. Participants needed to have complete covariate information

Table S4. Individual characteristics of the analysis sample, by experimental group

	Control (*n* = 913)	MTL (*n* = 907)	N-S (*n* = 924)	WL (*n* =895)	PC (*n* = 891)	Overall (*n* = 4,530)
	***n* (%)**	***n* (%)**	***n* (%)**	***n* (%)**	***n* (%)**	***n* (%)**
**Sex**						
Female	518 (57)	512 (56)	534 (58)	510 (57)	512 (57)	2,586 (57)
Male	395 (43)	395 (44)	390 (42)	385 (43)	379 (43)	1,944 (43)
**Age**						
18-29	61 (7)	65 (7)	56 (6)	56 (6)	41 (5)	279 (6)
30-39	139 (15)	101 (11)	141 (15)	132 (15)	129 (15)	642 (14)
40-49	169 (19)	181 (20)	171 (19)	180 (20)	180 (20)	881 (19)
50-59	199 (22)	204 (23)	187 (20)	185 (21)	194 (22)	969 (21)
60-69	179 (20)	191 (21)	197 (21)	181 (20)	185 (21)	933 (21)
70+	166 (18)	164 (18)	172 (19)	160 (18)	161 (18)	823 (18)
**Ethnicity**						
White British	799 (88)	789 (87)	809 (88)	792 (88)	785 (88)	3,974 (88)
White other	53 (6)	55 (6)	51 (6)	50 (6)	50 (6)	259 (6)
Mixed or multiple ethnic groups	9 (1)	13 (1)	15 (2)	6 (1)	11 (1)	54 (1)
Asian or Asian British	37 (4)	33 (4)	28 (3)	32 (4)	25 (3)	155 (3)
Black or Black British	12 (1)	14 (2)	17 (2)	13 (1)	16 (2)	72 (2)
Other	3 (0)	3 (0)	4 (0)	2 (0)	4 (0)	16 (0)
**Education**						
Degree or equivalent +	460 (50)	435 (48)	450 (49)	426 (48)	425 (48)	2,196 (48)
A levels or vocational level 3 or equivalent	174 (19)	174 (19)	179 (19)	174 (19)	177 (20)	878 (19)
Other qualifications below A levels or equivalent	163 (18)	155 (17)	167 (18)	158 (18)	141 (16)	784 (17)
Other qualification	46 (5)	70 (8)	44 (5)	49 (6)	62 (7)	271 (6)
No qualifications	70 (8)	73 (8)	84 (9)	88 (10)	86 (10)	401 (9)
**Children in household**						
Yes	280 (31)	262 (29)	269 (29)	286 (32)	280 (31)	1,377 (30)
No	633 (69)	645 (71)	655 (71)	609 (68)	611 (69)	3,153 (70)
**Shopping responsibility**						
Yes – some or all	868 (95)	866 (95)	895 (97)	859 (96)	852 (96)	4,340 (96)
No – someone else does	45 (5)	41 (5)	29 (3)	36 (4)	39 (4)	190 (4)
**Current label use**						
Very often	177 (19)	209 (23)	179 (19)	220 (25)	160 (18)	945 (21)
Quite often	297 (33)	345 (38)	305 (33)	308 (34)	291 (33)	1,546 (34)
Occasionally	269 (29)	234 (26)	267 (29)	266 (30)	282 (32)	1,318 (29)
Rarely	132 (14)	95 (10)	142 (15)	83 (9)	107 (12)	559 (12)
Never	38 (4)	24 (3)	31 (3)	18 (2)	51 (6)	162 (4)
**Reported consuming or buying product in past 12 months**						
Pizza	670 (73)	647 (71)	686 (74)	690 (77)	668 (75)	3,361 (74)
Drink	313 (34)	336 (37)	324 (35)	336 (38)	321 (36)	1,630 (36)
Cake	627 (69)	651 (72)	633 (69)	635 (71)	617 (69)	3,163 (70)
Crisps	752 (82)	739 (82)	772 (84)	753 (84)	707 (79)	3,723 (82)
Yoghurt	761 (83)	755 (83)	772 (84)	747 (84)	744 (84)	3,779 (83)
Breakfast cereal	779 (85)	757 (84)	755 (82)	756 (85)	755 (85)	3,802 (84)
**Currently trying to lose weight**						
Yes	416 (46)	428 (47)	464 (50)	411 (46)	406 (46)	2,125 (47)
No	457 (50)	450 (50)	434 (47)	449 (50)	450 (51)	2,240 (49)
Prefer not to say	40 (4)	29 (3)	26 (3)	35 (4)	35 (4)	165 (4)
	**Control (*n* = 913)**	**MTL (*n* = 907)**	**N-S (*n* = 924)**	**WL (*n* =895)**	**PC (*n* = 891)**	**Overall (*n* = 4,530)**
**Interested in healthy eating**						
Very interested	342 (37)	408 (45)	415 (45)	363 (41)	366 (41)	1,894 (42)
Quite interested	483 (53)	451 (50)	448 (49)	478 (53)	472 (53)	2,332 (51)
Not very interested	83 (9)	45 (5)	54 (6)	48 (5)	50 (6)	280 (6)
Not at all interested	5 (1)	3 (0)	7 (1)	6 (1)	3 (0)	24 (1)
**Knowledge in healthy eating^**						

A lot of knowledge	218 (24)	277 (31)	274 (30)	251 (28)	255 (29)	1,275 (28)
Some knowledge	549 (60)	543 (60)	515 (56)	530 (59)	492 (55)	2,629 (58)
A little knowledge	140 (15)	85 (9)	127 (14)	109 (12)	141 (16)	602 (13)
No knowledge	6 (1)	2 (0)	7 (1)	5 (1)	3 (0)	23 (1)

MTL, Multiple Traffic Light; N-S, Nutri-Score; WL, Warning Label; PC, Positive Choice tick; RR: Relative Risk; CI: Confidence Interval.

Table S5. Summary of participants who correctly ranked the healthiest product at baseline and follow-up, by FOPL group and product category

	**Control**		**MTL**		**N-S**		**WL**		**PC**		**Overall**	
	**Baseline**	**Follow-up**	**Baseline**	**Follow-up**	**Baseline**	**Follow-up**	**Baseline**	**Follow-up**	**Baseline**	**Follow-up**	**Baseline**	**Follow-up**
	***n* (%)**	***n* (%)**	***n* (%)**	***n* (%)**	***n* (%)**	***n* (%)**	***n* (%)**	***n* (%)**	***n* (%)**	***n* (%)**	***n* (%)**	***n* (%)**
**Pizza**	537 (80)	531 (79)	547 (85)	604 (93)	575 (84)	656 (96)	557 (81)	592 (86)	565 (85)	574 (86)	2,781 (83)	2,957 (88)
**Drinks**	222 (71)	213 (68)	232 (69)	315 (94)	213 (66)	312 (96)	237 (71)	286 (85)	218 (68)	244 (76)	1,122 (69)	1,370 (84)
**Cake**	518 (83)	505 (81)	543 (83)	609 (94)	529 (84)	603 (95)	509 (80)	576 (91)	507 (82)	491 (80)	2,606 (82)	2,784 (88)
**Crisps**	546 (73)	549 (73)	556 (75)	695 (94)	569 (74)	739 (96)	565 (75)	668 (89)	530 (75)	542 (77)	2,766 (74)	3,193 (86)
**Yoghurt**	134 (18)	130 (17)	134 (18)	657 (87)	114 (15)	669 (87)	120 (16)	483 (65)	87 (12)	197 (27)	589 (16)	2,136 (57)
**Cereal**	384 (49)	370 (48)	354 (47)	678 (90)	354 (47)	684 (91)	339 (45)	577 (76)	349 (46)	409 (54)	1,780 (47)	2,718 (72)

MTL, Multiple Traffic Light; N-S, Nutri-Score; WL, Warning Label; PC, Positive Choice tick. Needed to consume the product to be included

Table S6. Multiple regression analysis results- association between time taken to rank products at follow-up and FOPL group compared to control - without requirement for being correct (adjusted for baseline ranking time, device used, design factors and covariates)

	**MTL v Control RM (95%CI)**	**N-S v Control RM (95%CI)**	**WL v Control RM (95%CI)**	**PC v Control RM (95%CI)**	**N-S v MTL RM (95% CI)**
**Pizza**	1.21 (1.15, 1.28) **P < 0.001**	0.99 (0.94, 1.05) 0.779	1.22 (1.16, 1.29) **P < 0.001**	1.02 (0.96, 1.07) 0.591	0.82 (0.77, 0.87) **P < 0.001**
**Drink**	1.35 (1.25, 1.44) **P < 0.001**	1.08 (1.00, 1.16) 0.045	1.27 (1.18, 1.36) **P < 0.001**	1.05 (0.98, 1.13) 0.182	0.80 (0.75, 0.86) **P < 0·001**
**Cake**	1.40 (1.32, 1.48) **P < 0.001**	1.15 (1.09, 1.22) **P < 0.001**	1.54 (1.46, 1.63) **P < 0.001**	1.08 (1.02, 1.15) 0.008	0.82 (0.78, 0.87) **P < 0·001**
**Crisps**	1.39 (1.32, 1.45) **P < 0.001**	1.13 (1.08, 1.18) **P < 0.001**	1.28 (1.22, 1.34) **P < 0.001**	1.14 (1.08, 1.19) **P < 0.001**	0.81 (0.78, 0.85) **P < 0·001**
**Yoghurt**	1.26 (1.20, 1.33) **P < 0.001**	1.03 (0.97, 1.08) 0.321	1.23 (1.17, 1.29) **P < 0.001**	1.01 (0.96, 1.06) 0.788	0.81 (0.77, 0.86) **P < 0·001**
**Cereal**	1.25 (1.19, 1.31) **P < 0.001**	0.99 (0.95, 1.05) 0.846	1.32 (1.25, 1.38) **P < 0.001**	1.00 (0.95, 1.06) 0.867	0.80 (0.76, 0.84) **P < 0·001**

All analyses were adjusted for the five stratification factors (year of recruitment to panel, sex, age, government office region, household income) and the following pre-specified covariates: ethnicity, highest education level, household composition, food shopping responsibility, current FOPL use, baseline ranking time and device used. Participants needed to have complete covariate information and buy/eat cereal/pizza to be included. MTL: Multiple Traffic Lights; N-S: Nutri-Score; WL: Warning label; PC: Positive Choice tick.

Table S7. Mean global food score and standard deviations by experimental group and equivalised household income per month – without requirement of buying/eating all food products

	**Control (*n* = 913) mean (SD)**	**MTL (*n* = 907) mean (SD)**	**N-S (*n* = 924) mean (SD)**	**WL (*n* =895) mean (SD)**	**PC (*n* = 891) mean (SD)**	**Overall (*n* = 4,530) mean (SD)**
**Equivalised income per month**						
More than £2000	0.0 (0.7)	2.1 (1.3)	2.1 (1.3)	1.5 (1.4)	0.2 (0.9)	1.2 (1.4)
£1251- £2000	-0.0 (0.8)	2.2 (1.2)	2.1 (1.4)	1.5 (1.4)	-0.0 (0.8)	1.2 (1.5)
£801-£1250	-0.1 (0.9)	1.8 (1.3)	1.8 (1.4)	1.4 (1.4)	0.1 (0.9)	1.0 (1.5)
£800 or less	0.1 (0.7)	1.3 (1.5)	2.1 (1.5)	1.0 (1.5)	0.1 (0.9)	0.9 (1.5)

Participants needed to have full co-variate information to be included. MTL: Multiple Traffic Lights; N-S: Nutri-Score; WL: Warning label; PC: Positive Choice tick; SD: standard deviation.
